# Mathematiklehrveranstaltung neu – digital und invertiert

**DOI:** 10.1007/978-3-658-31279-4_22

**Published:** 2020-07-08

**Authors:** Marc Peterfi, Manfred Daniel

**Affiliations:** 1 Duale Hochschule Baden-Württemberg, Karlsruhe, Germany; 2 ILIAS open source e-Learning e.V., Köln, Germany; 3 Technische Hochschule Ostwestfalen-Lippe, Lemgo, Germany; 4 Duale Hochschule Baden-Württemberg, Karlsruhe, Germany; Duale Hochschule Baden-Württemberg Karlsruhe, Karlsruhe, Deutschland

## Abstract

Studierende im ersten Semester stehen in MINT-Fächern vor der Herausforderung, den Übergang von der Schulmathematik zur Hochschulmathematik zu meistern. Im Projekt optes wird das Ziel verfolgt, die Studierenden bei diesem Übergang zu unterstützen. Dieser betrifft nicht nur die Zeit vor der Aufnahme des Studiums, sondern im Besonderen auch die ersten Semester in einem Studiengang mit mathematischen Lehranteilen.

## 21.1 Einleitung

Studierende im ersten Semester stehen in MINT-Fächern vor der Herausforderung, den Übergang von der Schulmathematik zur Hochschulmathematik zu meistern. Im Projekt optes wird das Ziel verfolgt, die Studierenden bei diesem Übergang zu unterstützen. Dieser betrifft nicht nur die Zeit vor der Aufnahme des Studiums, sondern im Besonderen auch die ersten Semester in einem Studiengang mit mathematischen Lehranteilen. Das vorliegende Konzept der digitalen Mathematiklehrveranstaltung hat zum Ziel, die Studierenden beim Erlernen mathematischer Inhalte auch im Studium weiter zu unterstützen. Im Portfolio der optes-Angebote schlägt die digitale Mathematiklehrveranstaltung die Brücke zwischen den Vorkursangeboten der Studieneingangsphase und den summativen Prüfungen durch E-Klausuren. Doch wie kann man die Genese mathematischer Kompetenzen fördern und unterstützen? Innerhalb des optes-Teilprojekts „E-Assessment im Studium" wird hierfür ein Ansatz mit einer möglichst aktiven Präsenz und einer Unterstützung des Übens durch elektronisches Assessment verfolgt.

*Rahmenbedingungen*

Zunächst sollen die spezifischen Rahmenbedingungen des beschriebenen Versuchs dargestellt werden. Die Herausforderungen und Anforderungen eines Übungsbetriebes sind in großer Weise vom zeitlichen Umfang (SWS), der Gruppengröße und der Hochschulart abhängig. Entwickelt wurde die Lehrveranstaltung mit dem Gedanken einer Anwendung an der Dualen Hochschule Baden-Württemberg (DHBW) Karlsruhe im grundständigen Studiengang Wirtschaftsinformatik. Das bedeutet jedoch nicht, dass dieses Konzept nicht auch in anderen Studiengängen oder an anderen Hochschularten eingesetzt werden kann. Auf diese Möglichkeit wird am Ende eingegangen werden. An der DHBW werden im Studiengang Wirtschaftsinformatik die Studierenden eines Jahrganges auf fünf Kurse mit einer jeweiligen Größe von 30 bis 35 Studierenden aufgeteilt. Die Kurseinteilung und der soziale Kursverbund bleiben im Regelfall für das gesamte Studium bestehen. Ein wichtiger Aspekt ist, dass für die Studierenden im dualen Studium aufgrund ihrer Arbeitsverhältnisse bei den dualen Partnern in den Präsenzveranstaltungen Anwesenheitspflicht herrscht.

In den meisten Fällen werden die Module in den verschiedenen Kursen von jeweils verschiedenen Lehrenden gehalten. Lehrende sind an der Dualen Hochschule hauptamtliche Professor*innen oder externe Lehrbeauftragte. Im vorliegenden Fall des Jahrganges 2019 gab es für das Teilmodul „Analysis und Lineare Algebra“ in den fünf Kursen vier Dozent*innen. Unter diesen Lehrenden wurde im vorliegenden Fall ein gemeinsames Curriculum anhand der Modulbeschreibung vereinbart und eine inhaltlich einheitliche Klausur über alle Kurse erstellt. Dennoch war jeder im Sinne der Wissenschafts- und Lehrfreiheit in der Ausgestaltung seiner Lehrveranstaltung im Rahmen der Modulbeschreibung frei. Diese sieht für das Teilmodul 30 Unterrichtstunden Präsenz und 45 Unterrichtsstunden Selbststudium vor. Ebengleiches trifft auch für das Teilmodul „Logik und Algebra“ des zweiten Semesters zu. Das Gesamtmodul umfasst damit 5 ECTS-Punkte. Die beiden Teilmodule eines dieser Kurse werden im vorliegenden Konzept umgesetzt und betreffen einen Kurs mit 33 Studierenden.

## 21.2 Das Grundkonzept: Inverted Classroom

Das bisherige didaktische Modell der Mathematiklehrveranstaltungen an der DHBW sieht seminaristische Vorlesungen im kleinen Kursverband mit integrierten Übungsphasen vor. Im betrachteten Studiengang werden ausnahmsweise freiwillig zu besuchende Tutorien angeboten. Das Selbststudium wird nicht explizit durch die Lehrperson gestaltet oder betreut.

Der Fokus lag bei der Entwicklung der digitalen Mathematiklehrveranstaltung in der Unterstützung des Übungsprozesses. Dabei sollte sowohl das individuelle Lernen und Üben als auch die gegenseitige Unterstützung der Studierenden bei der Bearbeitung von Übungsaufgaben gefördert werden. Außerdem sollte die Lehrperson besser befähigt werden, die Defizite der Studierenden zu erfassen und ihnen zielgerichtet dabei zu helfen, diese zu überwinden. In vielen klassischen mathematischen Lehrbetrieben schließen die Übungseinheiten an eine frontal durchgeführte Vorlesung an. Während die Lehrperson in der Vorlesung durch Vortrag den Stoff an die Studierenden vermittelt, wird die Übung und Rekapitulation des Stoffes und seiner Anwendungen in nachgelagerte Übungseinheiten, wie Tutorien und Saalübungen, oder ins Selbststudium ausgelagert. Je nach Größe und verfügbarer Zeit einer Mathematiklehrveranstaltung eines Studienganges kann diese nur aus Übungsblättern und Musterlösungen bis hin zu Saalübungen und Tutorien durch Lehrkräfte oder studentische Hilfskräfte reichen. Diesen Lösungen ist gemein, dass eine gegenseitige Rückmeldung zwischen Lehrenden und Studierenden, so es diese überhaupt geben kann, erst mit zeitlicher Verzögerung möglich ist. Aus diesen Beweggründen wurde die Lehrveranstaltung im sogenannten *Inverted Classroom Model* (ICM) (Schäfer 2012; Handke 2012; Lage, Platt und Treglia 2000; siehe auch Kapitel 20) bzw. dem *Inverted Classroom Mastery Model* (Handke

2013) umgesetzt. In diesen ist die Stofferarbeitung in ein Selbststudium vorgelagert, während die Vorteile der Anwesenheit der Lehrperson in den Präsenzlehreinheiten zur aktiven Besprechung der Inhalte und Probleme und zur Durchführung von Übungen genutzt werden kann. Die Lehrperson kann dabei im direkten Kontakt mit den Studierenden auf die Schwierigkeiten des aktuellen Themas eingehen. Um eine solche Präsenzsitzung optimal vorzubereiten, bietet es sich an, die Lernmaterialien der Studierenden, die diese zur Stofferarbeitung nutzen können, durch interaktive Fragestellungen anzureichern. Dabei sollen diese sowohl den Studierenden zur direkten Selbstreflexion als auch der Lehrperson zur optimalen Vorbereitung der Präsenz dienen. Zusätzlich wird die Präsenz durch kurze Einreichaufgaben vorbereitet. Im Gegensatz zur oben geschilderten Vorgehensweise der der Präsenz nachgelagerten Übungseinheiten sind die Ergebnisse damit bereits vor der Präsenzsitzung bekannt. Damit kann die Lehrperson im Optimalfall die Implikationen aus den Testergebnissen zur Vorbereitung der Sitzung nutzen, und die Besprechung fällt in die Sitzung, die sowieso der Vertiefung und Übung des Stoffes gewidmet ist. In diesem Kontext bekommt der Einsatz elektronischer Lernmaterialien und Übungsaufgaben einen besonderen Sinn.

## 21.3 Lernmaterial und Tests im Selbststudium

Die Lernplattform ILIAS stellt nach Ansicht der Autoren geeignete Mittel für diese Bedarfe zur Verfügung.

### *21.3.1 Lernmodule*

Zur Stofferarbeitung wurden den Studierenden die mathematischen Inhalte eines Themas in dem ILIAS-Objekt „Lernmodul“ (siehe auch Kapitel 15) aufbereitet zur Verfügung gestellt. Diese interaktiven Lehrbücher ermöglichen nicht nur die einfache Verlinkung eines Inhaltes auf eine andere Seite oder eine Fortschrittsanzeige, sondern die Studierenden erhalten zu vielen Aufgaben direkte Rückmeldungen durch das genutzte Lernmanagementsystem. Jene Rückmeldungen erhalten die Studierenden an Ort und Stelle. Damit sind das Aufschlagen von Lösungsseiten und Vergleichen der Lösung, welche man von Übungsbüchern kennt und die ein potenzielles Motivationshemmnis sein können, nicht nötig. Auch ist der Vergleich von Musterlösungen mit der eigenen Antwort von studentischer Seite aus möglicherweise fehlerbelastet. Zu den Fragen folgt zusätzlich auch eine erklärende Musterlösung, die auch ohne Beantwortung der Frage aufrufbar ist.

Die Abbildung 1 zeigt eine Frage innerhalb eines Lernmoduls. Die Studierenden erhalten eine direkte Rückmeldung, ob sie die Frage korrekt beantwortet haben. Zudem steht ihnen eine Übersicht des Bearbeitungsstandes der Lernmodulseiten zur Verfügung. Existiert auf einer Seite eine falsch beantwortete Frage,

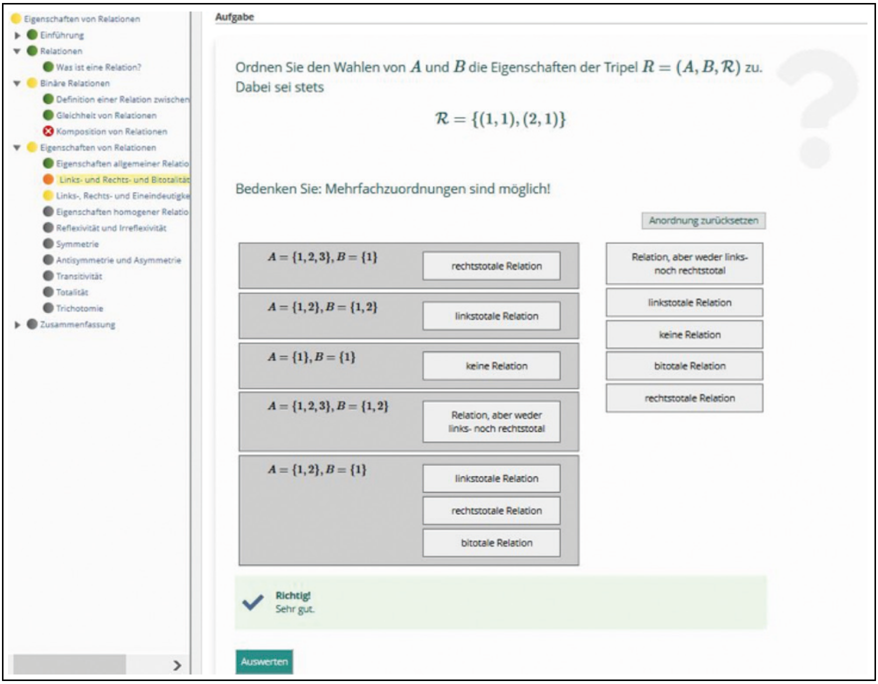

**Abbildung 1:** Frage in einem Lernmodul der Lehrveranstaltung „Logik und Algebra“

so wird diese mit einem roten Kreis samt Kreuz markiert. Teilweise bearbeitete Inhalte sind gelb, vollständig und korrekt bearbeitete Inhalte grün angezeigt (siehe auch Kapitel 17).

Neben der direkten Rückmeldung für die Studierenden erhält aber auch die Lehrperson eine Information darüber, wie häufig die jeweiligen Fragen beantworten und wie oft sie im ersten oder zweiten Anlauf korrekt gelöst wurden oder wie viele Studierende sogar mehr Versuche benötigten bzw. die Frage nie korrekt beantworten konnten. Die Rückschlüsse, die sich daraus ziehen lassen, können im besten Fall dafür genutzt werden, Verständnislücken der Gesamtheit der Studierenden des Kurses zu detektieren.

### *21.3.2 Präsenzvorbereitende Tests*

Eine weitere Vorbereitungsmaßnahme für die Präsenzsitzungen sind die präsenzvorbereitenden Tests. Dabei handelt es sich meist um kurze Tests, die Verständnislücken aufdecken sollen. Außerdem sollen diese Tests Lehrenden bei der Entscheidung helfen, ob und wie ein gewisses Thema vorbereitet werden soll.

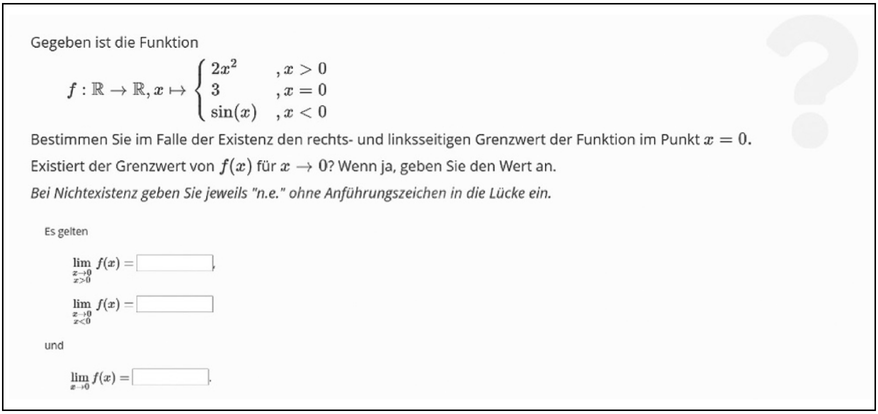

**Abbildung 2:** Erste Aufgabenstellung

So erhielten die Studierenden beispielsweise im Themenbereich Stetigkeit und Funktionsgrenzwertbestimmung zwei vergleichbare Fragen (siehe Abbildung 2 und Abbildung 3), deren Unterschied aber eine konzeptionelle Verständnislücke der Studierenden offenbaren konnte (Peterfi 2020). Dazu wurde eine abschnittsweise definierte Funktion erstellt. Gefragt wurde nach dem Funktionsgrenzwert in einer speziellen Stelle. Die Funktion war dabei so konzipiert, dass sie in dieser Stelle eine Unstetigkeit aufwies. Durch Ersetzung des Funktionswertes an dieser Stelle durch den Funktionsgrenzwert in dieser Stelle wäre die Funktion jedoch stetig. Viele der am Test teilnehmenden Studierenden konnten bei dieser Aufgabenstellung diesen Grenzwert jedoch nicht bestimmen oder übersprangen die Aufgabe.

Im Anschluss wurde der Funktionswert an der betreffenden Stelle durch eine Unbekannte ersetzt und die Studierenden erhielten die Aufgabe, die Unbekannte so zu bestimmen, dass die Funktion stetig war. Diese Variation der Aufgabe wurde von allen am Test teilnehmenden Studierenden korrekt beantwortet, obwohl es sich prinzipiell um die gleiche Aufgabe, die Bestimmung des Funktionsgrenzwertes, handelte. Daraus konnte der Rückschluss gezogen werden, dass das Konzept der Folgenstetigkeit von den Studierenden noch nicht verstanden war und ein vermutlich fehlerhaftes Verständnis des Stetigkeitsbegriffes angewandt wurde. Zudem war die Anzahl der Antworten bei der zweiten Frage höher. Eine mögliche Interpretation durch die Lehrperson war in diesem Fall, dass die Studierenden vor dem Begriff des Grenzwertes zurückschreckten oder diesen Inhalt nicht hinreichend bearbeitet hatten. Für die folgende Sitzung war somit der Bedarf dafür, nochmals auf Grenzwerte und ihre Zusammenhänge zur Stetigkeit einzugehen, naheliegend.

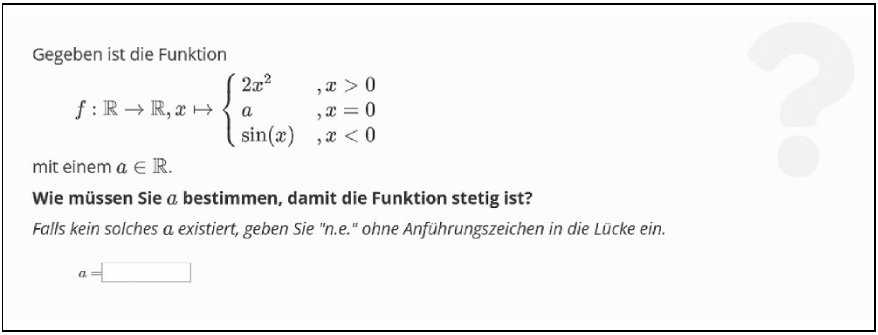

**Abbildung 3:** Zweite Aufgabenstellung

Im Gegensatz zu den ebenfalls angebotenen und automatisch ausgewerteten Trainings enthalten die präsenzvorbereitenden Tests auch manuell durch die Lehrperson zu bewertende Aufgaben. So können Freitext- oder Zeichenaufgaben eingesetzt werden. Die Lehrperson kann hier auch direkte schriftliche Rückmeldungen zu den Studierendenantworten geben. Da die Testeinstellungen in der digitalen Mathematiklehrveranstaltung auf anonym gestellt sind, ist das auch möglich, ohne dass die Lehrperson wissen muss, auf wessen Einreichung sie antwortet. Die Entscheidung, die Tests anonym durchzuführen, wurde unter anderem auch deshalb getroffen, da dies nach Aussage der Kursteilnehmer*innen die Hemmnisse einer Teilnahme an den Tests reduzierte.

### *21.3.3 Präsenzsitzungen*

Die Bearbeitung der interaktiven Lernmodule und präsenzvorbereitenden Tests soll in der Selbststudiumsphase vor der zugehörigen Präsenzsitzung stattfinden. Da dafür mindestens ein ähnlicher Zeitaufwand wie für eine Frontalsitzung eingeplant werden sollte, sind längere Übungsaufgaben in der Präsenz zu bearbeiten. Da die aktive Beteiligung und die gegenseitige Unterstützung der Studierenden untereinander primäre Zielsetzungen des Konzeptes sind, wurden vor allem im ersten Semester verschiedene aktivierende Methoden zwischen Übungsaufgaben eingesetzt. Beispielsweise wurden Phasen des aktiven Plenums (Spannagel und Spannagel 2013) eingebaut.

Für das zweite Semester wurde aufgrund der dabei gemachten Erfahrungen die Entscheidung getroffen, die Präsenzsitzung in einen aktiven Besprechungsteil und einen Übungsteil aufzuteilen. Im ersten Teil werden zunächst Fragen zu den Inhalten der Lernmodule und präsenzvorbereitenden Tests beantwortet. Außerdem kann die Lehrperson in dieser Phase auf die Dinge eingehen, die ihr in der Vorbereitung der Sitzung bei Begutachtung der Ergebnisse der Fragen in den Lernmodulen und Tests aufgefallen sind. Für diese erste Phase ist ein Drittel der Präsenzveranstaltung eingeplant. Im zweiten Drittel der jeweils drei Unterrichtsstunden langen Präsenzsitzungen wird dann primär durch Bearbeitung von Übungsaufgaben geübt. Die Aufgaben werden den Studierenden auch hier über die ILIAS-Lernplattform zur Verfügung gestellt. Hilfreich ist, dass es sich um duale Studierende handelt, die zumeist bereits mit entsprechenden Endgeräten in der Lehrveranstaltung arbeiten. Nicht selten handelt es sich dabei auch um die von den dualen Partnern als Arbeitgeber der Studierenden zur Verfügung gestellten Arbeitsgeräte. Sollte während der Bearbeitung der Übungsaufgaben der Bedarf einer gemeinsamen Besprechung eines Themas durch den gesamten Kurs aufkommen, so kann die Übungseinheit hierfür durch eine aktive Besprechung unterbrochen werden.

Der Vorteil der Nutzung der digitalen Übungsangebote in der Präsenz liegt in mehreren Punkten.

- Zunächst können die elektronischen Aufgaben in ILIAS mit gestuft aufrufbaren Lösungshinweisen ausgestattet werden und liefern zudem direkte automatische Rückmeldungen, was die Lehrperson von einigen Fragen entlastet. Außerdem können die Lösungsvorschläge in den hier genutzten Testeinstellungen durch die Studierenden direkt in der Frage aufgerufen werden. Das ermöglicht trotz einer Gruppengröße von 33 Studierenden, dass die Lehrperson ausreichend Zeit hat, um durch die Reihen zu gehen und auf dann immer noch offene Fragen individuell einzugehen.
- Da das Üben zudem in der Präsenz stattfindet, besteht für die Studierenden die Möglichkeit der gemeinsamen Bearbeitung von Aufgaben. Damit steigt grundsätzlich auch die verfügbare Zeit, die Studierende mit anderen Studierenden zusammen an Übungsaufgaben arbeiten können. Das gilt im Besonderen auch für Studierende mit längeren Anreisezeiten zur Studienakademie.
- Ein positiver Nebeneffekt dieser Nutzung digitaler Übungsaufgaben auch in der Präsenz zeigte sich im Verlauf der Lehrveranstaltung „Logik und Algebra", als aufgrund der Präventionsmaßnahmen im Rahmen von Covid-19 der Präsenzbetrieb eingestellt werden musste. Die Lehrveranstaltung konnte (Stand März 2020) dennoch mit nur minimalen Einschränkungen weiterlaufen. In der vorbereitenden Selbststudiumsphase gab es keine Änderungen im Ablauf zur Veranstaltung vor der Krise. Aber auch viele Vorteile der Präsenz konnten aufgrund dieses Übungskonzeptes beibehalten werden.

### *21.3.4 Freier Trainingsplatz*

Zudem wurde den Studierenden ein Freier Trainingsplatz (Schmitt 2020) zur Verfügung gestellt. Dabei handelt es sich um einen ILIAS-Test im Continous Testing Mode. Ein Test in diesem Modus erlaubt der Lehrperson, anders als die übrigen Tests, nachträglich Aufgaben zum Test hinzuzufügen oder zu entfernen, ohne die Testergebnisse der Studierenden zu löschen. Damit konnte ein zentraler Bereich für freiwillige Übungsaufgaben geschaffen werden, der nach und nach mit aktuellen Inhalten befüllt werden konnte. Der Freie Trainingsplatz erlaubt zudem den Studierenden die Nutzung von Taxonomiefiltern zum Erstellen verschiedener individueller Fragenauswahlen. Hier wurde den Studierenden „Taxonomien nach Inhalt“ zur Verfügung gestellt. Auch konnten die Studierenden bestimmte Methoden, zum Beispiel Aufgaben zu der Regel von de l’Hospital oder speziellen Integrationsregeln, gezielt auswählen. Auch eine vorgefertigte Taxonomie für spezielle Trainings wurde zur Verfügung gestellt. Insgesamt können sich damit Studierende Tests nach eigenen Kriterien zusammenstellen und zielgerichtet an Schwachstellen arbeiten. Leider wird eine einmal korrekt beantwortete Frage vom Freien Trainingsplatz nicht erneut vorgelegt. Daher konnte dieser von einigen Studierenden nicht mehr für die direkte Prüfungsvorbereitung genutzt werden. Aus diesem Grund wurde für die Lehrveranstaltung „Logik und Algebra“ entschieden, den Freien Trainingsplatz erst im Rahmen der Prüfungsvorbereitung zur Verfügung zu stellen. In dieser Phase kann er seine Stärken des zielgerichteten Übens spezieller Themen voll entfalten. Im Rahmen der Evaluation der Lehrveranstaltung „Analysis und Lineare Algebra“ gaben 75% der an der Umfrage teilnehmenden Studierenden an, den Freien Trainingsplatz genutzt zu haben. Außerdem gaben fast 30% an, diesen mehrmals pro Woche genutzt zu haben. Fast die Hälfte der Teilnehmer*innen der Evaluation gaben hingegen an, den Freien Trainingsplatz fast nie bzw. nie genutzt zu haben.

### *21.3.5 Elektronische Klausur und elektronische Probeklausur*

Der formative Einsatz von E-Assessment in der digitalen Mathematiklehrveranstaltung wurde in der Nutzung einer elektronischen Klausur (siehe Kapitel 11 und Kapitel 12) auch summativ fortgeführt. Die Studierenden waren durch die elektronischen Tests während der Lehrveranstaltung bereits mit den Aufgabentypen der E-Klausur vertraut. Dieser Aussage wurde in der Evaluation zugestimmt. Zusätzlich wurde mit allen Studierenden eine elektronische Probeklausur durchgeführt, die in der Evaluation von den Studierenden positiv bewertet wurde.

## 21.4 Lessons Learned und Evaluation

Die Evaluation der Lehrveranstaltung „Analysis und Lineare Algebra“ wurde in Zusammenarbeit mit dem an der Universität Hamburg angesiedelten optes-Teilprojekt „Prozessbegleitung und didaktische Beratung“ (siehe Kapitel 25) durchgeführt. Dazu wurden nach Ende des letzten Veranstaltungstermins Online-Umfragen durchgeführt. Dabei lagen von den 33 Studierenden des mit der digitalen Mathematiklehrveranstaltung (im folgenden ICM-Kurs) durchgeführten Kurses nach Bereinigung Daten von 28 Studierenden vor. Das entspricht einer Quote der ausgewerteten Daten von knapp 85% der Grundgesamtheit im ICM-Kurs. Eine

Vergleichsumfrage in den vier parallel stattfindenden Kursen (im folgenden Vergleichsgruppe) lieferte nach Bereinigung Daten von 52 Studierenden. Damit liegt die Quote in der Vergleichsgruppe im Bereich um 40%. Zur Auswertung wurden Mittel der deskriptiven Statistik gewählt. Im Folgenden stehe M für den Mittelwert und SD für die Standardabweichung. Die Teilnehmenden der Umfrage konnten ihre Zustimmung zu Aussagen über die Lehrveranstaltung auf einer fünfstufigen Likert-Skala geben. Diese reichte dabei von 1 für „stimme voll und ganz zu“ bis 5 für „stimme nicht zu“. Außerdem hatten die Teilnehmenden die Möglichkeit, die Option „keine Angabe / trifft auf mich nicht zu“ auszuwählen. Einige der Fragen ließen dabei Vergleichsmöglichkeiten zwischen dem ICM-Kurs und der Vergleichsgruppe zu.

Im Rahmen der Umfrage wurde so auch nach der Mathematiknote in der Hochschulzugangsberechtigung gefragt. Bei einem Durchschnittswert von 2,18 (SD=0,50) über beide Teilnehmergruppen lag diese beim ICM-Kurs im Mittel bei 2,35 (SD=1,73) und in der Vergleichsgruppe bei 2,10 (SD=0,96). Allerdings sind diese Zahlen aufgrund des geringen Teilnehmeranteils an den Umfragen in der Vergleichsgruppe störanfällig. Es ist nicht auszuschließen, dass in den Umfragen der Vergleichsgruppe Studierende mit besonders guten oder besonders schlechten Vorleistungen nicht teilgenommen haben.

Ein späterer Vergleich der Ergebnisse der Teilmodulprüfung mit den Lehrenden von drei der anderen vier Kurse zeigte, dass Mittelwert und Median der Noten im ICM-Kurs jedoch besser lagen als in diesen parallelen Kursen. Gemittelt über alle Teilnehmenden dieser drei Kurse und des ICM-Kurses lag der Notenschnitt bei 2,9. Dahingegen lag die Durchschnittsnote im ICM-Kurs bei 2,2. Der Durchschnitt der drei verglichenen Kurse lag zusammengenommen bei 3,2. Auch der Median des ICM-Kurses (2,0) lag besser als in den verglichenen Parallelkursen.

Jedoch sind diese Ergebnisse durch verschiedene Einflüsse überlagert. So wurde die Prüfung im ICM-Kurs auf elektronische Weise durchgeführt, während die anderen Kurse eine inhaltlich identische klassische Klausur auf Papier durchführten. Die Korrektur der Ergebnisse geschah in jedem Kurs durch die zugehörige Lehrperson. Eine Vergleichbarkeit kann damit aufgrund der Konstellation nicht als gesichert gelten. Weiter sind die Prüfungsnoten im Studiengang auch aus Erfahrung nicht gleichmäßig über alle Kurse verteilt. Trotz dieser statistischen Störfaktoren erscheint das bessere Abschneiden des ICM-Kurses in der Klausur beachtenswert.

Die Evaluation der ersten Lehrveranstaltung „Analysis und Lineare Algebra“ zeigte, dass die Lehrperson aus Sicht der Studierenden in der invertierten Lehrveranstaltung eher auf die Fragen der Teilnehmenden eingehen konnte (M=1,41/1,95) und die Studierenden sich bei der Strukturierung des Lernprozesses besser unterstützt gefühlt hatten (M=2,28/2,97). Darüber hinaus konnten in der Evaluation des ersten Durchlaufes keine wesentlichen Unterschiede in Fragen der Unterstützung zu den parallelen Kursen festgestellt werden. Die Evaluation konnte außerdem leider nicht bestätigen, dass die Studierenden sich in der Präsenzphase aktiver fühlten. Der Arbeitsaufwand wurde in der invertierten Lehrveranstaltung deutlich höher eingestuft als in der Vergleichsgruppe. Darauf wurde in der folgenden Lehrveranstaltung „Logik und Algebra“ reagiert.

Zur Qualitätskontrolle wird am Ende jedes Lernmoduls der Lehrveranstaltung „Logik und Algebra“ auf eine kurze Umfrage weitergeleitet. In dieser haben die Studierenden die Möglichkeit, auf Umfang, Verständlichkeit und Zeitaufwand einzugehen und persönliche Bemerkungen abzugeben. Zum Zeitpunkt der Erstellung dieses Berichts waren Ergebnisse zu drei Lernmodulen verfügbar. Die Ergebnisse dieser Kurzumfragen lassen darauf deuten, dass die Studierenden den Umfang der Lernmodule als angemessen betrachten. Außerdem herrscht Zustimmung zur Aussage, dass die Fragen in den Lernmodulen hilfreich seien. Allerdings haben an diesen Umfragen, auf die man mit einem Link am Ende eines Lernmoduls gelangt, lediglich 9 bzw. 8 der 33 Studierenden teilgenommen. Damit kann nicht ausgeschlossen werden, dass lediglich motivierte Studierende an den Umfragen zu den Lernmodulen teilgenommen haben. Hier ist auf die Ergebnisse der Abschlussevaluation nach Ende der Lehrveranstaltung zu warten.

## 21.5 Nutzbarkeit der digitalen Mathematiklehrveranstaltung in anderen Kontexten

Das Lehrveranstaltungskonzept wurde auf die Rahmenbedingungen des Studiengangs Wirtschaftsinformatik an der Dualen Hochschule Baden-Württemberg Karlsruhe angepasst entwickelt. Dennoch können die Lernmodule, Fragen und die Veranstaltungskonzeption grundsätzlich auch in anderen Studiengängen oder an anderen Hochschularten eingesetzt werden. Dazu sollen die Lernmodule und Fragenpools ebenso wie eine Handreichung zur Durchführung und Anpassbarkeit der digitalen Mathematiklehrveranstaltung, wie bei in optes erstellten Materialien üblich, unter einer Creative-Commons-Lizenz veröffentlicht werden. Dabei ist das Konzept am besten zu nutzen, wenn vergleichbare Gruppengrößen vorliegen. Die Begrenzung der Teilnehmerzahl liegt darin begründet, dass die Lehrperson auf die Studierenden eingehen soll. Dafür muss in der Präsenzveranstaltung genug Zeit für individuelles Feedback an die Studierenden vorhanden sein. Auch die Auswertung der Fragen hinsichtlich Verständnislücken zur Präsenzvorbereitung geschieht durch die Lehrperson persönlich und nicht durch ein automatisches System. Die digitale Mathematiklehrveranstaltung erlaubt diese Betreuung der Studierenden durch die Lehrperson für diese Gruppengröße, ohne einen zu großen Zeitaufwand auf Seite der Lehrperson und der Studierenden zu verlangen. Damit kann sie auch dann eingesetzt werden, wenn keine Ressourcen für studentische Hilfskräfte wie beim in optes ebenfalls verfolgten E-Mentoring (siehe Kapitel 18) zur Verfügung stehen. Bei höheren Gruppengrößen kann über den Einsatz eines Übungsbetriebes mit mehreren Lehrpersonen und damit einer Aufteilung der Studierenden in Gruppen mit überschaubarer Größe nachgedacht werden. Die Lernmodule und automatisch korrigierten Tests können auch außerhalb dieses Szenarios angewandt werden.
